# FIGNL1 Inhibits Non-homologous Chromosome Association and Crossover Formation

**DOI:** 10.3389/fpls.2022.945893

**Published:** 2022-07-11

**Authors:** Shuying Yang, Chao Zhang, Yiwei Cao, Guijie Du, Ding Tang, Yafei Li, Yi Shen, Hengxiu Yu, Zhukuan Cheng

**Affiliations:** ^1^Jiangsu Co-Innovation Center for Modern Production Technology of Grain Crops, Yangzhou University, Yangzhou, China; ^2^State Key Lab of Plant Genomics, Institute of Genetics and Developmental Biology, Innovation Academy for Seed Design, Chinese Academy of Sciences, Beijing, China; ^3^College of Advanced Agricultural Sciences, University of Chinese Academy of Sciences, Beijing, China

**Keywords:** FIGNL1, non-homologous recombination, crossover, meiosis, rice

## Abstract

Meiotic crossovers (COs) not only generate genetic diversity but also ensure the accuracy of homologous chromosome segregation. Here, we identified FIGNL1 as a new inhibitor for extra crossover formation in rice. The *fignl1* mutant displays abnormal interactions between non-homologous chromosomes at diakinesis, and chromosome bridges and fragmentation at subsequent stages of meiosis, but shows normal homologous chromosome pairing and synapsis during early prophase I. FIGNL1 participates in homologous chromosome recombination and functions downstream of DMC1. Mutation of *FIGNL1* increases the number of bivalents in *zip4* mutants, but does not change the number of HEI10 foci, indicating that FIGNL1 functions in limiting class II CO formation. FIGNL1 interacts with MEICA1, and colocalizes with MEICA1 in a dynamic pattern as punctate foci located between two linear homologous chromosomes. The localization of FIGNL1 depends on ZEP1-mediated assembly of the synaptonemal complex. Based on these results, we propose that FIGNL1 inhibits non-homologous chromosome interaction and CO formation during rice meiosis.

## Introduction

Meiosis is a cell division program unique to sexually propagating eukaryotes. Compared with mitosis, meiosis has one more round of cell division, leading to the formation of four gametes with halved genetic material. Several key events occur during this process, including homologous chromosome pairing, synapsis, and recombination (Kleckner, 1996; Zickler and Kleckner, 1999). These events are the foundation of stable meiosis and have received considerable attention. Meiotic crossovers (COs) are the products of homologous recombination (HR) and important for precise homologous chromosome separation in both plants and animals.

The production of double-strand breaks (DSBs) marks the beginning of HR (Szostak et al., 1983). SPO11, a protein which catalyzes the production of DSBs, is then removed by the MRX (Mre11-Xrs2-Rad50) complex (Bergerat et al., 1997; Keeney et al., 1997; Hamant et al., 2006). This complex is also responsible for the incision from the 5′ end of DSBs, exposing 3′-single-stranded DNA (Cannavo and Cejka, 2014; Marini et al., 2019). After that, RPA is recruited for the stabilization of the 3′-single-stranded DNA (Wang and Haber, 2004). RAD51 and DMC1 are yeast homologs of RecA (Shinohara et al., 2000). During the HR process, DMC1 plays a crucial role, while RAD51 is an accessory (Cloud et al., 2012). Before homology search and single strand invasion, RAD51 and DMC1 bind to the 3′-single-stranded DNA for the formation of nucleoprotein filaments (Brown et al., 2015). RAD51-DMC1 co-foci are often arranged in pairs separated by distances of up to 400 nm (Brown et al., 2015). After that, a crucial step of HR is that RAD51 and DMC1 mediate homology search and single strand invasion, leading to the formation of D-loops (Nara et al., 2001; Sauvageau et al., 2005; Pradillo et al., 2012; Ines et al., 2013). D-loops are precursors of COs and non-crossovers (NCOs). NCOs are produced at a much higher rate than COs (Torlazzi et al., 1995). Formation of NCOs mainly occurs through the synthesis-dependent strand annealing (SDSA) (McMahill et al., 2007). During SDSA, the extended break end is displaced from the D-loop and annealed to the complementary sequence at the non-invading break end (Elbakry et al., 2021). If a CO has formed between a pair of homologous chromosomes, the possibility that a CO forms nearby is decreased (Otto and Payseur, 2019). This phenomenon is called CO interference. Based on their sensitivity to the interference, COs are further classified into class I COs and class II COs (Li et al., 2021). ZMM proteins are responsible for generating class I COs, which are also referred to as interference-sensitive COs (Zhang et al., 2019). In *Arabidopsis thaliana*, the production of class II COs relies on MUS81 and other endonucleases (Santos et al., 2003; Berchowitz et al., 2007; Higgins et al., 2008).

Only a small portion of recombination intermediates are processed as COs, leading to the restricted number of COs between each pair of homologous chromosome (Mercier et al., 2015). This phenomenon suggests the existence of mechanisms inhibiting the production of COs. Three pathways inhibiting the production of COs have been revealed in *Arabidopsis*, including FANCM (and some other factors, such as MHF1 and MHF2), the FIGL1-FLIP complex, and the RECQ4-Top3α-RMI1 (RTR) complex (Fernandes et al., 2018). FANCM was reported to be involved in the dissolution of the D-loop into NCOs by promoting the SDSA pathway (Crismani et al., 2012; Lorenz et al., 2012; Girard et al., 2014). The FIGL1-FLIP complex was suggested to limit the production of COs by inhibiting the homology search and single strand invasion mediated by RAD51 and DMC1 (Fernandes et al., 2018). The RTR complex was reported to resolve double Holliday junction (dHJ) into NCOs (Knoll et al., 2014; Seégueéla-Arnaud et al., 2015). Although these studies have revealed several mechanisms for limiting the production of COs in *Arabidopsis*, only a few such proteins have been identified in rice.

Our previous studies revealed that MEICA1 is an inhibitor of meiotic COs in rice (Hu et al., 2017). MSH5 is a member of ZMM proteins that are responsible for class I CO formation (Luo et al., 2013). The number of bivalents in the *msh5* background is increased by the absence of MEICA1 (Hu et al., 2017). The *meica1* mutant displayed evident meiosis defects, including interactions between non-homologous chromosomes, chromosome bridges, and chromosome fragmentation. Based on these phenotypes and the interaction of MEICA1 with MSH7, MEICA1 was suggested to have a function in preventing abnormal recombination during the process of DSB repairing.

Here, we identified FIGNL1, which forms a conserved complex with MEICA1 in many species (Hu et al., 2017), as a new inhibitor of meiotic COs. We found that the *fignl1* mutant has meiotic defects similar to those of *meica1*, including associations between non-homologous chromosomes, indicating that FIGNL1 may have a similar function in preventing abnormal recombination during DSB repair. *fignl1* has been reported to exhibit multivalent chromosomes at diakinesis, lagging chromosomes, and chromosome fragments during meiosis, showing that FIGNL1 is involved in rice meiosis (Zhang et al., 2017). However, its role in CO regulation has not been revealed. Because of the associations between non-homologous chromosomes in *fignl1* meiocytes, it is difficult to count the number of COs according to the shape of bivalents or multivalents. Therefore, we took the advantage of cytological and genetic analyses to explore the role of FIGNL1 in controlling CO formation. The lack of FIGNL1 increased the number of bivalents in *zip4*, but had no effect on the number of HEI10 foci, indicating that FIGNL1 is an inhibitor of class II COs. Genetic analyses revealed that FIGNL1 acts downstream of DMC1. However, DMC1 foci disappeared later in *fignl1* than in wild type, suggesting FIGNL1 may control the temporal distribution of DMC1. On the basis of these findings, together with the interaction of FIGNL1 with RAD51 and DMC1, we propose that FIGNL1 is likely to inhibit the homology search and single strand invasion mediated by RAD51 and DMC1 to reduce the production of COs, just like its homolog FIGL1 in *Arabidopsis* (Fernandes et al., 2018). Therefore, we conclude that FIGNL1 may prevent abnormal recombination and limit CO formation during rice meiosis.

## Materials and Methods

### Plant Materials

*fignl1* was identified from Yandao 8 (a *japonica* rice variety) induced by ^60^Co-γ ray radiation. The *fignl1*-cas9 mutant was obtained from Nipponbare (a *japonica* rice variety) edited through CRISPR-Cas9 technology. Other reported mutants including *p31^comet^*, *ku70*, *dmc1*, *zip4* were also used in our study. Nipponbare was used as the wild type. All rice plants were cultivated in paddy fields in Beijing during the summer season.

### Map-Based Cloning of *fignl1*

*fignl1*^+^ *fignl1*^–^ plants were crossed with the *indica* rice Zhongxian 3037 to generate the mapping populations. 242 sterile F2 progeny and 493 sterile F3 progeny plants were obtained to conduct map-based cloning. InDel markers were designed according to sequence differences between 9311 and Nipponbare provided by NCBI. All the marker sequences are showed in Supplementary Table 1.

### CRISPR-Cas9 Targeting of *FIGNL1*

The CRISPR-Cas9 target sequence of *FIGNL1* was chosen online by CRISPR-PLANT, a portal of CRISPR-Cas9 mediated genome editing. The target sequence of *FIGNL1* was introduced into *Aar* I digested intermediate vector SK-gRNA. Then the vector was digested with *Kpn* I and *BamH* I to recycle the fragment. The vector pC1300-cas9 was also digested with *Kpn* I and *BamH* I to recycle the vector scaffold. Then the fragment containing the target sequence and the part of SK-gRNA was ligated with pC1300-cas9 scaffold to generate the final vector (Wang et al., 2015). The final vector was transformed into Yandao 8 mediated by Agrobacterium strain EHA105.

### Meiotic Chromosome Preparation

Both the wild-type and the mutant panicles at meiotic stages were collected and fixed in Carnoy’s solution (ethanol:glacial acetic, 3:1). Anthers were squashed with acetocarmine solution on slides and covered with coverslips. After that, they were washed with 45% acetic acid solution. Slides were soaked in liquid nitrogen for a short time. After removing the coverslips and dehydrating the slides through an ethanol gradient (70%, 90%, and 100%), the chromosomes were stained with 4,6-diamidino-phenylindole (DAPI) in an anti-fade solution (Vector Laboratories, Burlingame, CA). Chromosome images were captured under the Olympus BX61 fluorescence microscope with a micro CCD camera.

### Immunofluorescence

Fresh panicles of both the wild type and mutants at proper meiotic stages were chosen and fixed in 4% (w/v) paraformaldehyde for 30-50 min at room temperature. Anthers were squashed with 1 × PBS solution on slides and covered with coverslips. Before removing coverslips, slides were frozen in in liquid nitrogen. After that, they were dehydrated through an ethanol series (70%, 90% and 100%). Then slides were incubated with different antibodies diluted 1:50 in 1 × PBST solution for 1 h at 37 °C. After washing in 1 × PBS for 3 times, they were incubated with the second antibody for 30 min at 37 °C. After the second washing in 1 × PBS for three times, slides with chromosomes were stained with DAPI in an antifade solution (Vector Laboratories).

### Fluorescence *in situ* Hybridization

The FISH assay was performed according to a previous protocol (Cheng, 2013). The 5*S* ribosomal RNA probe was used to monitor states of homologous chromosome pairing.

### Yeast Two-Hybrid Assays

Young rice panicles of the wild type were collected to construct prey cDNA library. The full-length cDNA sequence of FIGNL1 was ligated with pGBKT7 to construct bait vector. The bait vector and prey cDNA library were used to conduct library screening with Matchmaker Library Construction & Screening kit (Clontech). Several full-length cDNA sequence and truncated sequence were cloned to pGADT7 and pGBKT7. All these vectors were transformed with Yeast Y2H Gold strain. The primer sequences used in Y2H are listed in Supplementary Table 1.

### Bimolecular Fluorescence Complementation Assays

To perform bimolecular fluorescence complementation (BiFC) assays, the full-length coding sequences of FIGNL1, MEICA1, RMI and MUS81 were independently introduced into pSCYNE (R) and pSCYCE(R). Rice stem protoplasts were cotransformed with plasmid pairs. SCFP3A signals were detected by a confocal laser scanning microscope.

### Split-Luciferase Complementation Assay

*Agrobacterium tumefaciens* containing vectors were injected into leaves of *Nicotiana benthamiana* and incubated for 24–48 h in the growth room. LUC activity was measured by CCD imaging system and luminometer. Protein interaction was estimated based on fluorescence intensity. Primers used in Split-Luciferase complementation assay are listed in Supplementary Table 1.

## Results

### Cloning of *FIGNL1*

**FIGURE 1:**
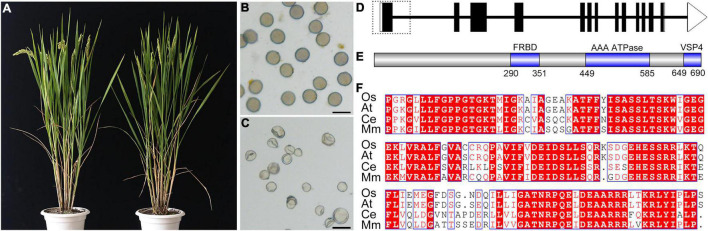
Characterization of the *fignl1* mutant and structure of the FIGNL1 protein. **(A)** Comparison of wild-type and *fignl1* plants. **(B)** Viable pollen grains of wild type. Bar, 50 μm. **(C)** Inviable pollen grains of the *fignl1* mutant. Bar, 50 μm. **(D)** Gene structure and mutation of *FIGNL1*. Coding regions are shown as black boxes. Untranslated regions are shown as black lines. Deleted region in the *fignl1* mutant is shown as a dotted box. **(E)** Protein structure of FIGNL1. **(F)** Multiple sequence alignment of the ATPase region. Os, *Oryza sativa*; At, *Arabidopsis thaliana*; Ce, *Caenorhabditis elegans*; Mm, *Mus musculus*.

To identify genes crucial for rice meiosis, we performed a genetic screen for sterile mutants. One of the mutants exhibited normal vegetative growth but was completely sterile (Figure 1A). I_2_-KI staining showed that pollen grains of the mutant were completely inviable (Figures 1B,C). The fertile and sterile F2 progeny of the heterozygous mutant exhibited a 3:1 segregation ratio, suggesting that the sterile phenotype is caused by a single recessive mutation. To identify the mutated gene, a map-based cloning strategy was carried out. The mutated gene was preliminarily mapped to an interval between 14.49 and 16.19 cM on chromosome 12, which corresponded to markers M6 and M8, respectively. However, PCR fragments could not be amplified in the mutant using marker M7 located at 15.16 cM on chromosome 12, indicating a deletion between markers M6 and M8 (Supplementary Figure 1). Using Tail-PCR, we identified a deletion of 308 kb including the site at 15.16 cM on chromosome 12. The deleted region contains the first exon of *FIGNL1* (LOC_Os12g25720), which is expected to have a function in meiosis.

To verify that the mutation of *FIGNL1* is responsible for the mutant phenotype, we generated *FIGNL1*-CAS9 plants. These plants had a meiotic chromosome phenotype similar to that of the original *fignl1* mutant (see below; Supplementary Figure 2). This result indicated that *FIGNL1* is the gene responsible for the mutant phenotype.

*FIGNL1* consists of 13 exons and 12 introns and encodes a 694-amino acid protein (Figure 1D). FIGNL1 was predicted to have an ATPase domain (Figure 1E), a VPS4 domain, and a sequence similar to the human FIGNL1’s RAD51 binding domain (FRBD) (Yuan and Chen, 2013; Zhang et al., 2017). Multiple sequence alignment revealed that FIGNL1 and its orthologs in other species are highly conserved within the ATPase region (Figure 1F).

### Non-homologous Chromosome Associations in *fignl1*

**FIGURE 2:**
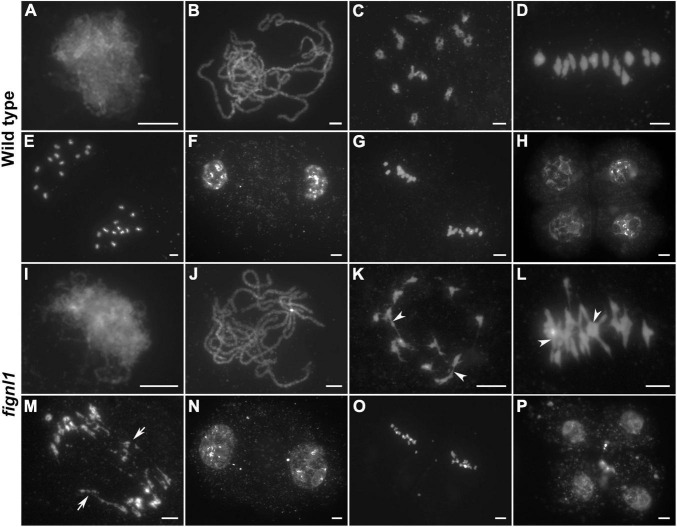
Meiotic chromosome behaviors of pollen mother cells in wild type and *fignl1*. Chromosomes were stained with DAPI. **(A,I)** Zygotene. **(B,J)** Pachytene. **(C,K)** Diakinesis. **(D,L)** Metaphase I. **(E,M)** Anaphase I. **(F,N)** Dyad. **(G,O)** Metaphase II. **(H,P)** Tetrad. Arrowheads point to some chromosome associations and arrows point to bridges. Scale bars, 5 μm.

To investigate whether the sterile phenotype of *fignl1* was caused by male meiosis defects, we used 4,6-diamidino-2-phenylindole (DAPI) staining to observe the meiotic chromosome behaviors in both wild-type and *fignl1* pollen mother cells (PMCs). In the wild type, chromosomes condensed to visible thin threads at leptotene. At zygotene, homologous chromosomes paired and started to synapse (Figure 2A). At pachytene, homologous chromosomes completed synaptonemal complex (SC) installation (Figure 2B). Thereafter, chromosomes continued to condense into 12 bivalents at diakinesis (Figure 2C). The 12 bivalents aligned on the equatorial plate at metaphase I (Figure 2D). During anaphase I and telophase I, homologous chromosomes separated from each other, then moved to opposite poles of the PMCs (Figure 2E). Meiosis I ended with the formation of dyads (Figure 2F). Then the dyads progressed through meiosis II, generating tetrads, which represented the completion of a round of meiosis (Figures 2G,H).

No obvious difference could be detected between the chromosomal behaviors of *fignl1* and wild type PMCs from zygotene to pachytene (Figures 2I,J). However, association among several bivalents was observed in *fignl1* PMCs at diakinesis, indicating abnormal interactions between non-homologous chromosomes (Figure 2K). At the same time, the homologous chromosomes of one bivalent were more tightly connected to each other in *fignl1* than those in the wild type. The abnormal association between non-homologous chromosomes became more frequent at metaphase I (Figure 2L). Among the 21 PMCs surveyed in *fignl1*, 20 meiocytes have chromosome bridges and fragments at anaphase I, giving rise to unequal chromosome segregation (Figure 2M). Finally, unequal chromosome segregation led to the formation of dyads and tetrads with micronuclei (Figures 2N–P). *FIGNL1*-CAS9 plants exhibited similar meiotic defects (Supplementary Figure 2). Therefore, we concluded that the sterile phenotype of *fignl1* resulted from unequal chromosome segregation.

We performed FISH assays to verify if the aberrant interactions were generated between random chromosomes in *fignl1* PMCs. 5*S* rDNA and the BAC clone OSJNBb0020J19 (J19) were chosen as available probes in these assays. 5*S* rDNA is a tandemly repetitive sequence that located near the centromere on chromosome 11 (Kamisugi et al., 1994), and the J19 is a chromosome 4-specific BAC clone in rice. We conducted FISH assays with these two probes in both wild type and *fignl1* metaphase I PMCs. 5*S* rDNA foci were observed on one bivalent (indicate chromosome 11) and the J19 foci were detected on another bivalent (indicate chromosome 4) in wild type. In *fignl1* PMCs from the same plant, one case was that 5*S* rDNA foci and J19 foci both existed on the same aberrant chromosome entanglement. Another case was that 5*S* rDNA foci and J19 foci were observed on two separated chromosomes, respectively (Supplementary Figure 3). According to our results, we speculated that the aberrant interactions between non-homologous chromosomes in *fignl1* occur randomly.

### FIGNL1 Is Dispensable for Homologous Chromosome Pairing and Synapsis

Considering that FIGNL1 plays an important role in preventing non-homologous chromosome interactions, we next wanted to explore whether FIGNL1 has any roles in homologous chromosome interactions. DAPI staining showed that homologous chromosome pairing and synapsis seemed to be unaffected by the loss of *FIGNL1* (Figure 2J).

**FIGURE 3:**
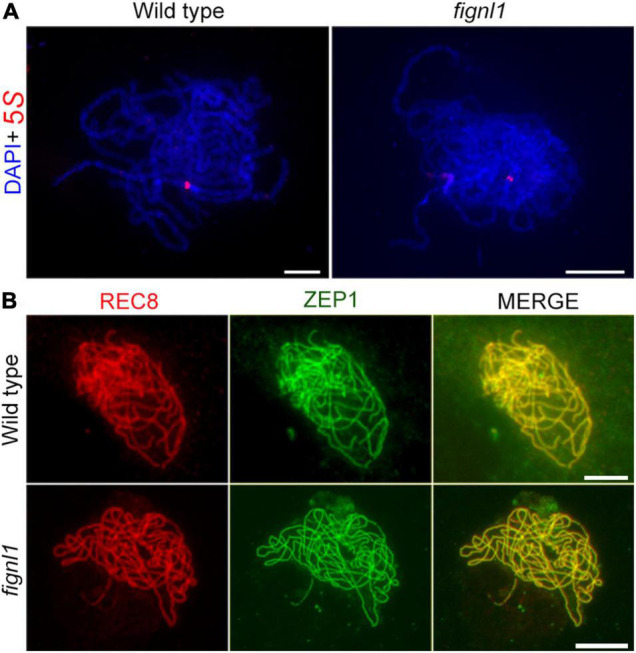
The mutation of *FIGNL1* has no effect on homologous chromosome pairing and synapsis. **(A)** FISH analysis with 5*S* rDNA probes (red) of the wild type and *fignl1* mutant. Chromosomes were stained with DAPI (blue). **(B)** Dual-immunolocalization of REC8 (red) and ZEP1 (green) in the wild type and *fignl1* mutant. Scale bars, 5 μm.

To explore the role of FIGNL1 in homologous chromosome pairing, we performed fluorescent *in situ* hybridization (FISH) with a 5*S* rDNA probe. The presence of two adjacent 5*S* rDNA signals at zygotene indicated that homologous chromosome pairing is normal in *fignl1* (Figure 3A). With the aim to determine if synapsis was completed in *fignl1*, we conducted immunolocalization analysis of ZEP1, which is the central element of the SC. At pachytene, no obvious difference was detected in the location of ZEP1 between *fignl1* and the wild type, indicating that homologous chromosome synapsis was unaffected in *fignl1* (Figure 3B). Based on these results, we concluded that FIGNL1 is not associated with homologous chromosome pairing and synapsis.

### FIGNL1 Is Involved in Homologous Chromosome Recombination and Functions Downstream of DMC1

**FIGURE 4:**
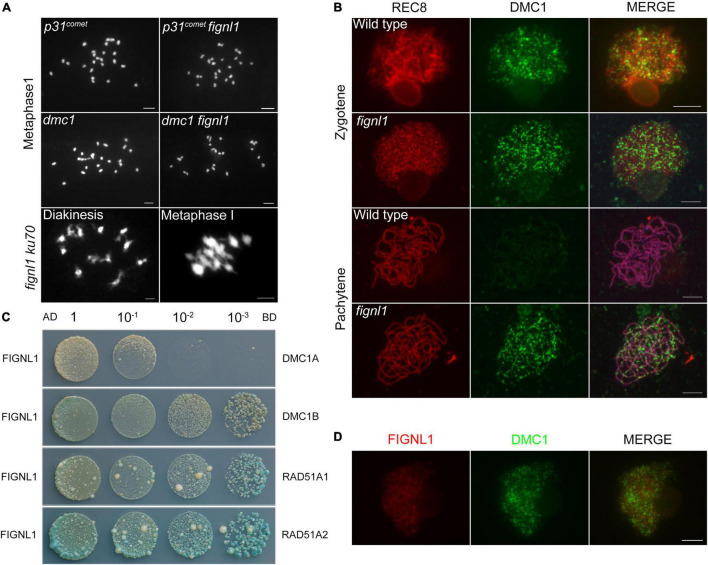
The relationship between FIGNL1 and DMC1. **(A)** Genetic analysis of FIGNL1 and DMC1. **(B)** Immunolocalization of REC8 (red) and DMC1 (green) in wild type and *fignl1.* **(C)** Examination of the interaction of FIGNL1 with RAD51A1, RAD51A2, DMC1A, and DMC1B by yeast two-hybrid assay. AD, prey vector; BD, bait vector. **(D)** Immunolocalization of FIGNL1 (red) and DMC1 (green) in wild type. Scale bars, 5 μm.

To explore whether the abnormal non-homologous chromosome associations in *fignl1* was dependent on meiotic DSB formation, we constructed the *p31*^comet^* fignl1* double mutant and performed cytogenetic analysis. Rice P31*^comet^* is involved in meiotic DSB formation (Ji et al., 2016). Owing to the lack of meiotic DSB formation, there are 24 univalents with a random distribution in the *p31^comet^* mutant. The meiotic phenotype of the *p31*^comet^* fignl1* double mutant was similar to that of the *p31^comet^* single mutant (Figure 4A). Therefore, we concluded that abnormal non-homologous chromosome associations in the *fignl1* mutant are dependent on meiotic DSB formation and that FIGNL1 functions downstream of P31*^comet^* during meiosis.

There are two main pathways involved in DSB repair: HR and classical non-homologous end joining (C-NHEJ) (Kakarougkas and Jeggo, 2014; Ceccaldi et al., 2015; Shibata, 2017). KU70 plays an important role in the C-NHEJ pathway (Deriano and Roth, 2013), while DMC1 is an essential factor in the HR pathway. DMC1 is present only in meiosis (Lan et al., 2020). To identify which meiotic DSB repair pathway FIGNL1 is associated with, the *ku70 fignl1* and *dmc1 fignl1* double mutants were generated. *ku70 fignl1* had a phenotype similar to that of *fignl1*, indicating that KU70 is not associated with the abnormal non-homologous chromosome interactions in *fignl1* (Figure 4A). On the other hand, the phenotype of *dmc1 fignl1* is similar to that of *dmc1*, which had almost 24 univalents (Figure 4A). Thus, we propose that the phenotype of *fignl1* is dependent on DMC1 and that FIGNL1 functions downstream of DMC1 during HR.

### FIGNL1 Regulates the Localization of DMC1 and Interacts With RAD51 and DMC1

To further confirm that FIGNL1 acts downstream of DMC1 during HR, we performed immunolocalization experiments. At zygotene, the localization of DMC1 in *fignl1* was undistinguishable from that in the wild type, suggesting that the loading of DMC1 is not affected by FIGNL1 (Figure 4B). Statistical analysis showed that the number of DMC1 foci in wild type (288.9 ± 8.645, *n* = 20) and that in *fignl1* mutants (290.5 ± 7.683, *n* = 20) did not have significant difference (Supplementary Figure 4). This finding supported the hypothesis that FIGNL1 functions downstream of DMC1. Nevertheless, we detected obvious DMC1 foci in *fignl1* at pachytene, when the DMC1 foci had already disappeared in the wild type, indicating a longer retention of DMC1 on chromosomes in *fignl1* than in the wild type (Figure 4B). The loading pattern of RAD51 is similar to that of DMC1 in both *fignl1* and the wild type (Supplementary Figure 5). We also found DMC1 foci existed on pachytene chromosomes in *meica1* (Supplementary Figure 6), just like those in *fignl1.* Since FIGNL1 regulates the temporal distribution of DMC1, we tried to verify whether they physically interacted. Yeast two-hybrid (Y2H) assays revealed that FIGNL1 interacts with RAD51A1, RAD51A2, DMC1A, and DMC1B (Figure 4C), which might account for the result that FIGNL1 has a function in regulating the loading of DMC1. Although FIGNL1 has a function in regulating the loading of DMC1, immunolocalization assays using antibodies against DMC1 and FIGNL1 revealed that FIGNL1 did not colocalize with DMC1 (Figure 4D).

### FIGNL1 Limits Meiotic Crossover Formation

**FIGURE 5:**
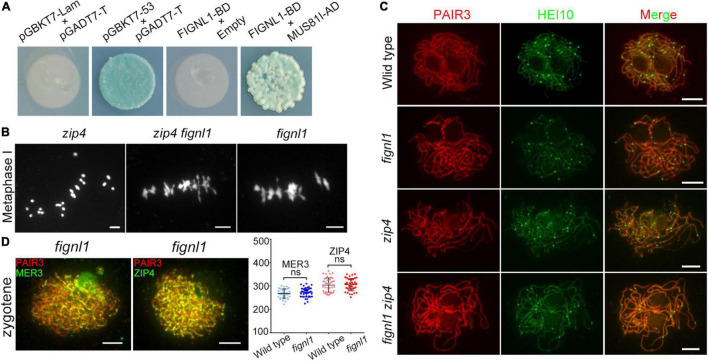
FIGNL1 limits CO formation. **(A)** FIGNL1 interacts with MUS81 in yeast two-hybrid assays. The interactions were detected by the growth and the blue color of yeast on QDO/X/A. AD, prey vector; BD, bait vector. **(B)** The mutation of *FIGNL1* restores bivalent formation in the *zip4* background. **(C)** Localization of HEI10 is independent of FIGNL1. **(D)** No significant differences were observed in the numbers of MER3 and ZIP4 foci between the wild type and *fignl1* mutant. Values are means ± SEM. ns, no significant difference according to two-tailed Student’s *t*-tests. Scale bars, 5 μm.

To explore the role of FIGNL1 during HR, a Y2H screen using the full-length FIGNL1 protein as the bait was conducted. As a result, FIGNL1 was found to interact with MUS81. Next, the full-length MUS81 coding sequence was cloned into the vector pGADT7, and the full-length FIGNL1 coding sequence was introduced into the vector pGBKT7. Yeast cells cotransformed with MUS81-AD and FIGNL1-BD grew normally on DDO and QDO/X/A plates, verifying the interaction between MUS81 and FIGNL1 (Figure 5A). BiFC assays further confirmed that FIGNL1 interacted with MUS81 *in vivo* (Supplementary Figure 7). Previous studies revealed that AtMUS81 participates in formation of class II COs (Berchowitz et al., 2007; Higgins et al., 2008). It is likely that FIGNL1 is involved in a related pathway to regulate CO formation in rice.

To explore whether FIGNL1 limits CO formation, we verified whether the bivalents in the *zmm* mutant could be restored by the mutation of *FIGNL1*. We conducted this assay instead of directly counting the chiasmata in *fignl1*, because the abnormal non-homologous chromosome interactions in *fignl1*. *zip4* is a *zmm* mutant with reduced bivalent formation (Shen et al., 2012). As expected, the number of bivalents in *zip4* was increased by the absence of FIGNL1, suggesting that FIGNL1 is an inhibitor of meiotic CO formation (Figure 5B).

The number of HEI10 foci at diakinesis is usually used as a marker monitoring the number of class I COs in rice (Wang et al., 2012; Li et al., 2018). To further explore the COs limited by FIGNL1 belong to class I or class II COs, we performed an immunofluorescence assay using antibodies against PAIR3 and HEI10. The number of HEI10 foci in *fignl1* (22.9 ± 0.4638, *n* = 40) was similar to that in the wild type (22.73 ± 0.3906, *n* = 40), indicating that FIGNL1 had no effect on the formation of class I CO (Figure 5C). Moreover, the number of HEI10 foci in *zip4* (16.13 ± 0.5595, *n* = 15) and that in *zip4 fignl1* (16.2 ± 0.4899, *n* = 15) did not have significant difference (Supplementary Figure 8). Together, these results suggested that FIGNL1 inhibits the formation of class II COs (Figure 5C). There was no significant difference between the number of MER3 foci (273 ± 3.183, *n* = 40) in *fignl1* and that in the wild type (268.4 ± 3.116, *n* = 40). The number of ZIP4 foci in *fignl1* (305.5 ± 4.13, *n* = 40) was undistinguishable from that in the wild type (304.1 ± 4.838 *n* = 40) (Figure 5D).

### FIGNL1 Is Located Between Homologous Chromosomes and Has a Dynamic Localization Pattern

Western blot analysis was used to test the specificity of the antibody against FIGNL1 (Supplementary Figure 9). However, we did not find any expected protein bands when the total panicle proteins of wild type were loaded in the blot. Considering that most proteins involved in meiosis are in the nucleus, we separated the nuclear and cytoplasmic fractions to obtain nuclear protein. Moreover, we used the antibody against FIGNL1 to immunoprecipitate FIGNL1 protein. As expected, a protein of the expected size was observed in western blots. We also collected the nuclear proteins from the *fignl1* mutant and used the antibody against FIGNL1 to conduct immunoprecipitation, but no protein of the expected size was observed in western blots. This result suggested the antibody against FIGNL1 is specific.

**FIGURE 6:**
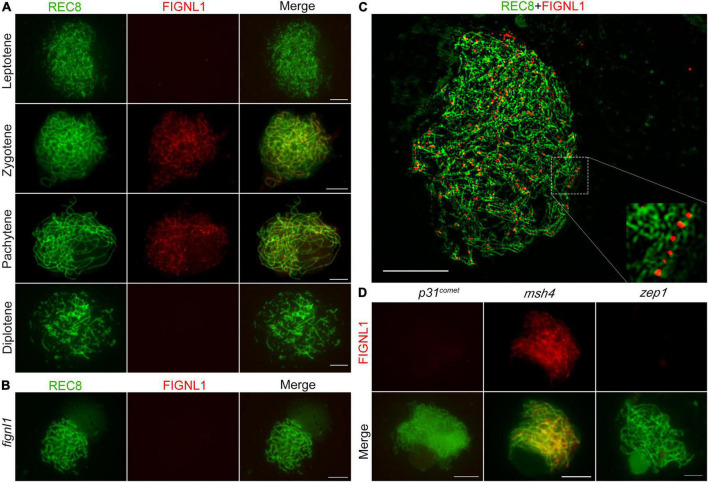
FIGNL1 is a meiotic chromatin-associated protein. **(A)** Dual immunolocalization of REC8 (green) and FIGNL1 (red) in wild-type meiocytes from leptotene to diplotene. **(B)** Dual immunolocalization of REC8 and FIGNL1 in *fignl1*. **(C)** FIGNL1 foci are located between two lines of REC8 signals. **(D)** Immunolocalization of FIGNL1 in *p31^comet^*, *msh4* and *zep1*. Bars, 5 μm.

To reveal the spatial and temporal distribution of FIGNL1 in meiocytes of the wild type, dual immunolocalization analysis was conducted with antibodies against REC8 and FIGNL1, which were raised in mouse and rabbit, respectively. Few FIGNL1 signals were observed in wild-type meiocytes at leptotene (Figure 6A). At zygotene, FIGNL1 signals were first visible as linear signals along meiotic chromosomes, and consisted of many punctate foci (Figure 6A). The punctate FIGNL1 foci persisted at pachytene and finally disappeared at diplotene (Figure 6A). To test whether the antibody against FIGNL1 is cytologically specific, immunolocalization was conducted in *fignl1* meiocytes. As expected, no FIGNL1 signal was detected (Figure 6B), further indicating that *fignl1* is a null mutant. To detect the precise localization of FIGNL1 foci on meiotic chromosomes, we observed the PMCs of the wild type at zygotene with a structured illumination microscope. The results showed that the FIGNL1 foci were located between two lines of REC8 signals (Figure 6C), which marked homologous chromosomes during meiosis. The spatial and temporal distributions of FIGNL1 suggested that FIGNL1 may play a role in processing DNA structures during meiotic recombination.

To investigate the function of FIGNL1 during HR, we next explored the effects of selected meiotic proteins on the localization of FIGNL1 by performing immunolocalization experiments in a set of HR-associated mutants (Figure 6D). P31*^comet^* is essential for meiotic DSB formation. No FIGNL1 signal was detected in *p31^comet^* mutants, suggesting that the localization of FIGNL1 is dependent on meiotic DSB formation. MSH4 is a member of ZMM protein family which are responsible for class I CO formation. The FIGNL1 signals in *msh4* meiocytes exhibited no significant difference from those in the wild type, indicating that the localization of FIGNL1 is not dependent on ZMM proteins. ZEP1 is the central element of the SC. There was no FIGNL1 signal in *zep1* meiocytes, implying that the loading of FIGNL1 depends on SC assembly.

### FIGNL1 Colocalizes With MEICA1

**FIGURE 7:**
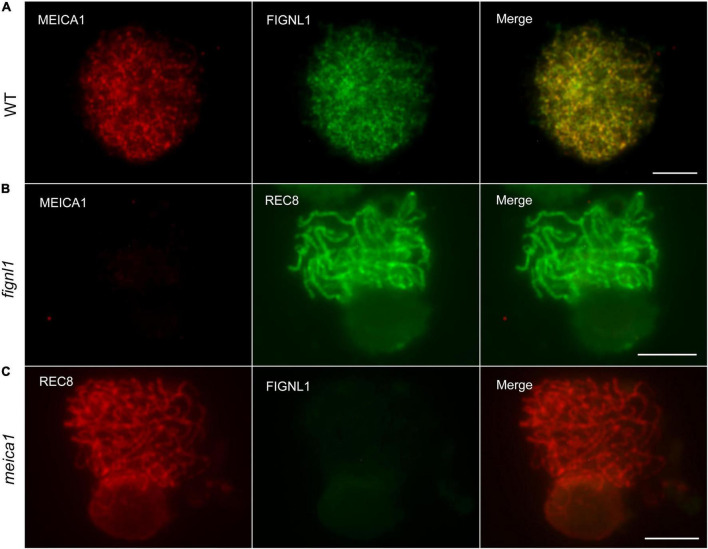
FIGNL1 colocalizes with MEICA1 and is required for the loading of MEICA1 (and *vice versa*). **(A)** FIGNL1 almost completely colocalizes with MEICA1 foci. **(B)** The immunodetection of MEICA1 in *fignl1*. **(C)** The immunodetection of FIGNL1 in *meica1*. Bars, 5 μm.

The loading pattern of FIGNL1 in the wild type is similar to that of MEICA1, whose ortholog in *Arabidopsis* has been reported to form a complex with FIGNL1. To investigate the relationship between FIGNL1 and MEICA1 in the wild type, dual immunolocalization analysis with antibodies against FIGNL1 and MEICA1 was conducted. As expected, the foci of FIGNL1 were almost completely colocalized with the foci of MEICA1 in all meiocytes examined (Figure 7A). No MEICA1 signal could be detected in *fignl1* meiocytes, implying that the loading of MEICA1 on meiotic chromosomes is dependent on the presence of FIGNL1 (Figure 7B). The loading of FIGNL1 on chromosomes also did not occur in the absence of MEICA1 (Figure 7C). Therefore, we concluded that both proteins depend on each other for chromosome localization.

### The Interaction Regions of FIGNL1 and MEICA1 Are Conserved

**FIGURE 8:**
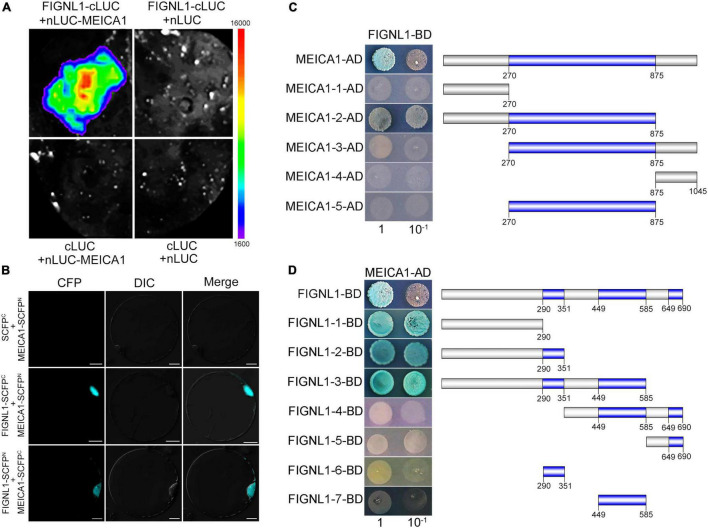
The region responsible for the interaction between FIGNL1 and MEICA1 is conserved. **(A)** FIGNL1 interacts with MEICA1 in LUC assays. **(B)** FIGNL1 interacts with MEICA1 in BiFC assays. **(C)** The region of MEICA1 responsible for the interaction between FIGNL1 and MEICA1. **(D)** The region of FIGNL1 responsible for the interaction between FIGNL1 and MEICA1.

In *Arabidopsis*, the orthologs of FIGNL1 and MEICA1 form a conserved complex that regulates HR. As expected, Y2H results showed that FIGNL1 interacts with MEICA1 (Supplementary Figure 10). The interaction was further confirmed by BiFC assay and split-luciferase complementation assay (Figures 8A,B). To further investigate the interaction region, truncated fragments of FIGNL1 and MEICA1 were introduced into the vectors pGBKT7 and pGADT7, respectively. We found that the N-terminal region and DUF4487 domain of MEICA1 and the N-terminal region of FIGNL1 are responsible for the interaction between FIGNL1 and MEICA1(Figures 8C,D). However, the interaction is not dependent on the three functional domains of FIGNL1, which is similar to what was observed in *Arabidopsis*, suggesting that the interaction regions are conserved.

## Discussion

### Functions of FIGNL1 in Meiosis

Loss of function of FIGNL1 causes obvious abnormal non-homologous chromosome associations and fragmentation. Although FIGNL1 affects non-homologous chromosome association, it has no effect on homologous chromosome pairing and synapsis. The phenotype of *fignl1* is similar to that of *meica1*, indicating that FIGNL1 may limit the association of non-homologous chromosomes by preventing non-allelic recombination in a similar way as MEICA1 (Hu et al., 2017).

We found that FIGNL1 is an inhibitor of meiotic CO formation using both genetic and cytological approaches. Three pathways inhibiting the production of COs have been revealed in *Arabidopsis*: FANCM (and some other factors, such as MHF1, MHF2), the FIGL1-FLIP complex, and the RTR complex (Fernandes et al., 2018). Both FANCM and the RTR complex have been reported to limit CO formation in rice. Because the plants with loss of *FANCM* or *RECQ4* are still fertile, molecular markers can be used to calculate recombination frequencies in these plants. The approach was used to reveal that FANCM and RECQ4 limit the formation of COs (Mieulet et al., 2018). However, since plants with loss of FIGNL1 were sterile, we could not use molecular markers to calculate the recombination frequency. We solved this problem using the cytological approach. We observed more bivalents in *zip4 fignl1* than in *zip4*, suggesting that FIGNL1 is an inhibitor of meiotic CO formation. The average number of HEI10 foci in *fignl1* was similar to that in the wild type, indicating that FIGNL1 has no effect on the formation of class I COs. Together these results suggested that FIGNL1 inhibits the formation of class II COs. Compared with *Arabidopsis* FIGL1, rice FIGNL1 not only has a common function in limiting class II CO formation, but also plays an additional important role in limiting non-homologous chromosome associations during the process of DSB repair.

### Mechanisms of FIGNL1 in Limiting Meiotic Crossovers

In rice, we found DMC1 begins to be loaded onto chromosomes at leptotene. Thereafter, FIGNL1 and MEICA1 bind to DMC1. At pachytene, DMC1 is gradually disassembled from chromosomes, leaving FIGNL1 and MEICA1 still be loaded on the chromosomes. MSH7, a protein reported to interact with MEICA1, prevents the formation of non-homologous chromosome associations and ensures accurate DSB repair. In the *fignl1* mutant, no MEICA1 foci were detected. Nevertheless, DMC1 remained on pachytene chromosomes, while they were disappeared during the similar stage in the wild type. The postponed disassembly of DMC1 from chromosomes may not only assure the homology search and single strand invasion between homologous chromosomes, but also give rise to abnormal recombination intermediates such as multichromatid joint molecules by aberrant strand invasion. These abnormal recombination intermediates could be processed as class II COs by several structure-specific endonucleases (Jessop and Lichten, 2008; Oh et al., 2008). MEICA1 has been reported to suppress non-homologous chromosome association. We found MEICA1 was not properly loaded on meiotic chromosomes in *fignl1*, resulting in abnormal non-homologous chromosome associations at diakinesis and metaphase I, and chromosome bridges and fragmentations in the subsequent stages of meiosis.

RAD51 and DMC1 foci remain on chromosomes longer in *fignl1* than in the wild type, suggesting that FIGNL1 may regulate the temporal distribution of DMC1 and is likely to promote the disassembly of DMC1. Y2H assays demonstrated the interaction of FIGNL1 with RAD51 and DMC1. FIGNL1 orthologs in humans and *Arabidopsis* have also been reported to interact with RAD51. This interaction is mediated by the FRBD domain of the FIGNL1 ortholog in humans. Previous studies suggested that the extra COs in *Arabidopsis figl1* arise from abnormal recombination intermediates produced by aberrant strand invasion (Girard et al., 2015). Thus, we propose that FIGNL1 is likely to play a similar role, promoting the disassembly of the RAD51 and DMC1 and ensuring the quality of homology search and single strand invasion, thus preventing the formation of abnormal recombination intermediates, including abnormal joint molecules and multichromatid joint molecules.

The assembly of RAD51/DMC1 is indispensable for HR, the disassembly of RAD51/DMC1 is also important for preventing the formation of toxic intermediates. Therefore, there must be a balance between the assembly and disassembly of RAD51/DMC1 at DSB sites, which is regulated by several functional proteins. A recent study revealed that *Arabidopsis* FIGL1 and BRCA2 have antagonistic functions in regulating RAD51 and DMC1 localization (Kumar et al., 2019). FIGL1 promotes the unloading of RAD51/DMC1 filaments and BRCA2 promotes the loading of RAD51/DMC1 filaments. The mutation of *FIGL1* restored RAD51/DMC1 foci and RAD51/DMC1-dependent synapsis in *brca2a brca2b* meiocytes. It is worth to explore the relationship between FIGNL1 and BRCA2 as well as their functions in regulating RAD51/DMC1 in rice. Considering that FIGNL1 interacts with RAD51 and DMC1 in both rice and Arabidopsis, we supposed that FIGNL1 may regulate the location of DMC1 by interacting with RAD51/DMC1. Although we proposed the increase of class II COs may arise from the inhibition of the homology search and single strand invasion mediated by RAD51 and DMC1, we cannot exclude the possibility that the abnormal non-homologous chromosome associations in *fignl1* also give rise to abnormal recombination intermediates, leading to the formation of class II COs.

## Data Availability Statement

The original contributions presented in the study are included in the article/Supplementary Material, further inquiries can be directed to the corresponding author/s.

## Author Contributions

ZC and HY conceived and designed the experiments. SY, CZ, YC, and GD performed the experiments. SY, CZ, YL, and YS analyzed the data. SY and ZC wrote the manuscript. All authors contributed to the article and approved the submitted version.

## Conflict of Interest

The authors declare that the research was conducted in the absence of any commercial or financial relationships that could be construed as a potential conflict of interest.

## Publisher’s Note

All claims expressed in this article are solely those of the authors and do not necessarily represent those of their affiliated organizations, or those of the publisher, the editors and the reviewers. Any product that may be evaluated in this article, or claim that may be made by its manufacturer, is not guaranteed or endorsed by the publisher.

## References

[B1] Berchowitz L. E. Francis K. E. Bey A. L. Copenhaver G. P. (2007). The role of AtMUS81 in interference-insensitive crossovers in *A. thaliana*. *PLoS Genet.* 3:e132. 10.1371/journal.pgen.0030132 PMC1941751 17696612

[B2] Bergerat A. de Massy B. Gadelle D. Varoutas P. C. Nicolas A. Forterre P. (1997). An atypical topoisomerase II from archaea with implications for meiotic recombination. *Nature* 386 414–417. 10.1038/386414a0 9121560

[B3] Brown M. S. Grubb J. Zhang A. Rust M. J. Bishop D. K. (2015). Small Rad51 and Dmc1 complexes often co-occupy both ends of a meiotic DNA double strand break. *PLoS Genet.* 11:e1005653. 10.1371/journal.pgen.1005653 26719980 PMC4697796

[B4] Cannavo E. Cejka P. (2014). Sae2 promotes dsDNA endonuclease activity within Mre11–Rad50–Xrs2 to resect DNA breaks. *Nature* 514 122–125. 10.1038/nature13771 25231868

[B5] Ceccaldi R. Rondinelli B. D’Andrea A. D. (2015). Repair pathway choices and consequences at the double-strand break. *Trends Cell Biol.* 26 52–64. 10.1016/j.tcb.2015.07.00 26437586 PMC4862604

[B6] Cheng Z. (2013). Analyzing meiotic chromosomes in rice. *Methods Mol. Biol.* 990 125–134. 10.1007/978-1-62703-333-6_13 23559209

[B7] Cloud V. Chan Y. L. Grubb J. Budke B. Bishop D. K. (2012). Dmc1 catalyzes interhomolog joint molecule formation in meiosis with Rad51 and Mei5-Sae3 as accessory factors. *Science* 337 1222–1225. 10.1126/science.1219379 22955832 PMC4056682

[B8] Crismani W. Girard C. Froger N. Pradillo M. Santos J. L. Chelysheva L. (2012). FANCM limits meiotic crossovers. *Science* 336 1588–1590. 10.1126/science.1220381 22723424

[B9] Deriano L. Roth D. B. (2013). Modernizing the nonhomologous end-joining repertoire: alternative and classical NHEJ share the stage. *Annu. Rev. Genet.* 47 433–455. 10.1146/annurev-genet-110711-155540 24050180

[B10] Elbakry A. Juhász S. Chan K. C. Löbricha M. (2021). ATRX and RECQ5 define distinct homologous recombination subpathways. *Proc. Natl. Acad. Sci. U.S.A.* 118:e2010370118. 10.1073/pnas.2010370118 33431668 PMC7826375

[B11] Fernandes J. B. Duhamel M. Seguéla-Arnaud M. Froger N. Girard C. Mercier R. (2018). FIGL1 and its novel partner FLIP form a conserved complex that regulates homologous recombination. *PLoS Genet.* 14:e1007317. 10.1371/journal.pgen.1007317 29608566 PMC5897033

[B12] Girard C. Chelysheva L. Choinard S. Froger N. Macaisne N. Lehmemdi A. (2015). AAA-ATPase FIDGETIN-LIKE 1 and helicase FANCM antagonize meiotic crossovers by distinct mechanisms. *PLoS Genet.* 11:e1005369. 10.1371/journal.pgen.1005369 26161528 PMC4498898

[B13] Girard C. Crismani W. Froger N. Mazel J. Lemhemdi A. Horlow C. (2014). FANCM-associated proteins MHF1 and MHF2, but not the other Fanconi anemia factors, limit meiotic crossovers. *Nucleic Acids Res.* 42 9087–9095. 10.1093/nar/gku614 25038251 PMC4132730

[B14] Hamant O. Ma H. Cande W. Z. (2006). Genetics of meiotic prophase I in plants. *Annu. Rev. Plant Biol.* 57 267–302. 10.1146/annurev.arplant.57.032905.105255 16669763

[B15] Higgins J. D. Buckling E. F. Franklin F. C. H. Jones G. H. (2008). Expression and functional analysis of AtMUS81 in *Arabidopsis* meiosis reveals a role in the second pathway of crossing-over. *Plant J.* 54 152–162. 10.1111/j.1365-313X.2008.03403.x 18182028

[B16] Hu Q. Li Y. Wang H. Shen Y. Zhang C. Du G. (2017). Meiotic chromosome association 1 interacts with TOP3α and regulates meiotic recombination in rice. *Plant Cell* 29 1697–1708. 10.1105/tpc.17.00241 28696221 PMC5559755

[B17] Ines O. D. Degroote F. Goubely C. Amiard S. Gallego M. E. White C. I. (2013). Meiotic recombination in *Arabidopsis* is catalysed by DMC1, with RAD51 playing a supporting role. *PLoS Genet.* 9:e1003787. 10.1371/journal.pgen.1003787 24086145 PMC3784562

[B18] Jessop L. Lichten M. (2008). Mus81/Mms4 endonuclease and Sgs1 helicase collaborate to ensure proper recombination intermediate metabolism during meiosis. *Mol. Cell* 31 313–323. 10.1016/j.molcel.2008.05.021 18691964 PMC2584117

[B19] Ji J. Tang D. Shen Y. Xue Z. Wang H. Shi W. (2016). P31comet, a member of the synaptonemal complex, participates in meiotic DSB formation in rice. *Proc. Natl. Acad. Sci. U.S.A.* 113 10577–10582. 10.1073/pnas.1607334113 27601671 PMC5035842

[B20] Kakarougkas A. Jeggo P. A. (2014). DNA DSB repair pathway choice: an orchestrated handover mechanism. *Br. J. Radiol.* 87:20130685. 10.1259/bjr.20130685 24363387 PMC4064598

[B21] Kamisugi Y. Nakayama S. Nakajima R. Ohtsubo H. Ohtsubo E. Fukui K. (1994). Physical mapping of the 5S ribosomal RNA genes on rice chromosome 11. *Mol. Gen. Genet.* 254 133–138. 10.1007/BF00283259 7816019

[B22] Keeney S. Giroux C. N. Kleckner N. (1997). Meiosis-specific DNA double- strand breaks are catalyzed by Spo11, a member of a widely conserved protein family. *Cell* 88 375–384. 10.1016/s0092-8674(00)81876-0 9039264

[B23] Kleckner N. (1996). Meiosis: how could it work? *Proc. Natl. Acad. Sci. U.S.A.* 93 8167–8174. 10.1073/pnas.93.16.8167 8710842 PMC38641

[B24] Knoll A. Schr0̈pfer S. Puchta H. (2014). The RTR complex as caretaker of genome stability and its unique meiotic function in plants. *Front. Plant Sci.* 5:33. 10.3389/fpls.2014.00033 24575106 PMC3921566

[B25] Kumar R. Duhamel M. Coutant E. Ben-Nahia E. Mercier R. (2019). Antagonism between BRCA2 and FIGL1 regulates homologous recombination. *Nucleic Acids Res.* 47 5170–5180. 10.1093/nar/gkz225 30941419 PMC6547764

[B26] Lan W. Lin S. Kao C. Chang W. Yeh H. Chang H. (2020). Rad51 facilitates filament assembly of meiosis-specific Dmc1 recombinase. *Proc. Natl. Acad. Sci. U.S.A.* 117 11257–11264. 10.1073/pnas.1920368117 32404423 PMC7260962

[B27] Li X. Zhang J. Huang J. Xu J. Chen Z. Copenhaver G. P. (2021). Regulation of interference-sensitive crossover distribution ensures crossover assurance in *Arabidopsis*. *Proc. Natl. Acad. Sci. U.S.A.* 118:e2107543118. 10.1073/pnas.2107543118 34795056 PMC8617516

[B28] Li Y. Qin B. Shen Y. Zhang F. Liu C. You H. (2018). HEIP1 regulates crossover formation during meiosis in rice. *Proc. Natl. Acad. Sci. U.S.A.* 115 10810–10815. 10.1073/pnas.1807871115 30275327 PMC6196533

[B29] Lorenz A. Osman F. Sun W. Nandi S. Steinacher R. Whitby M. C. (2012). The fission yeast FANCM ortholog directs non-crossover recombination during meiosis. *Science* 336 1585–1588. 10.1126/science.1220111 22723423 PMC3399777

[B30] Luo Q. Tang D. Wang M. Luo W. Zhang L. Qin B. (2013). The role of OsMSH5 in crossover formation during rice meiosis. *Mol. Plant* 6 729–742. 10.1093/mp/sss145 23220939

[B31] Marini F. Rawal C. C. Liberi G. Pellicioli A. (2019). Regulation of DNA double strand breaks processing: focus on barriers. *Front. Mol. Biosci.* 6:55. 10.3389/fmolb.2019.00055 31380392 PMC6646425

[B32] McMahill M. S. Sham C. W. Bishop D. K. (2007). Synthesis-dependent strand annealing in meiosis. *PLoS Biol.* 5:e299. 10.1371/journal.pbio.0050299 17988174 PMC2062477

[B33] Mercier R. Mézard C. Jenczewski E. Macaisne N. Grelon M. (2015). The molecular biology of meiosis in plants. *Annu. Rev. Plant Biol.* 66 297–327. 10.1146/annurev-arplant-050213-035923 25494464

[B34] Mieulet D. Aubert G. Bres C. Klein A. Droc G. Vieille E. (2018). Unleashing meiotic crossovers in crops. *Nat. Plants* 4 1010–1016. 10.1038/s41477-018-0311-x 30478361

[B35] Nara T. Hamada F. Namekawa S. Sakaguchi K. (2001). Strand exchange reaction in vitro and DNA-dependent ATPase activity of recombinant LIM15/DMC1 and RAD51 proteins from *Coprinus cinereus*. *Biochem. Biophys. Res. Commun.* 285 92–97. 10.1006/bbrc.2001.5095 11437377

[B36] Oh S. D. Lao J. P. Taylor A. F. Smith G. R. Hunter N. (2008). RecQ helicase, Sgs1, and XPF family endonuclease, Mus81-Mms4, resolve aberrant joint molecules during meiotic recombination. *Mol. Cell* 31 324–336. 10.1016/j.molcel.2008.07.006 18691965 PMC2587322

[B37] Otto S. P. Payseur B. A. (2019). Crossover interference: shedding light on the evolution of recombination. *Annu. Rev. Genet.* 53 19–44. 10.1146/annurev-genet-040119-093957 31430178 PMC8715713

[B38] Pradillo M. Lopez E. Linacero R. Romero C. Cunado N. Sanchez-Moran E. (2012). Together yes, but not coupled: new insights into the roles of RAD51 and DMC1 in plant meiotic recombination. *Plant J.* 69 921–933. 10.1111/j.1365-313X.2011.04845.x 22066484

[B39] Santos T. d. l. Hunter N. Lee C. Larkin B. Loidl J. Hollingsworth N. M. (2003). The Mus81/Mms4 endonuclease acts independently of double-Holliday junction resolution to promote a distinct subset of crossovers during meiosis in budding yeast. *Genetics* 164 81–94. 10.1093/genetics/164.1.81 12750322 PMC1462551

[B40] Sauvageau S. Stasiak A. Z. Banville I. Ploquin M. Stasiak A. Masson J. (2005). Fission yeast Rad51 and Dmc1, two efficient DNA recombinases forming helical nucleoprotein filaments. *Mol. Cell. Biol.* 25 4377–4387. 10.1128/MCB.25.11.4377-4387.2005 15899844 PMC1140613

[B41] Seégueéla-Arnaud M. Crismani W. Larchevêque C. Mazel J. Froger N. Choinard S. (2015). Multiple mechanisms limit meiotic crossovers: TOP3α and two BLM homologs antagonize crossovers in parallel to FANCM. *Proc. Natl. Acad. Sci. U.S.A.* 112 4713–4718. 10.1073/pnas.1423107112 25825745 PMC4403193

[B42] Shen Y. Tang D. Wang K. Wang M. Huang J. Luo W. (2012). ZIP4 in homologous chromosome synapsis and crossover formation in rice meiosis. *J. Cell Sci.* 125 2581–2591. 10.1242/jcs.090993 22393242

[B43] Shibata A. (2017). Regulation of repair pathway choice at two-ended DNA double-strand breaks. *Mutat. Res.* 803-805 51–55. 10.1016/j.mrfmmm.2017.07.011 28781144

[B44] Shinohara M. Gasior S. L. Bishop D. K. Shinohara A. (2000). Tid1/Rdh54 promotes colocalization of Rad51 and Dmc1 during meiotic recombination. *Proc. Natl. Acad. Sci. U.S.A.* 97 10814–10819. 10.1073/pnas.97.20.10814 11005857 PMC27106

[B45] Szostak J. W. Orr-Weaver T. L. Rothstein R. J. Stahl F. W. (1983). The double-strand-break repair model for recombination. *Cell* 33 25–35. 10.1016/0092-8674(83)90331-8 6380756

[B46] Torlazzi A. Xu L. Cao L. Kleckner N. (1995). Crossover and noncrossover recombination during meiosis: timing and pathway relationships. *Proc. Natl. Acad. Sci. U.S.A.* 92 8512–8516. 10.1073/pnas.92.18.8512 7667321 PMC41187

[B47] Wang C. Shen L. Fu Y. Yan C. Wang K. (2015). A simple CRISPR/Cas9 system for multiplex genome editing in rice. *J. Genet. Genomics* 42 703–706. 10.1016/j.jgg.2015.09.011 26743988

[B48] Wang K. Wang M. Tang D. Shen Y. Miao C. Hu Q. (2012). The role of rice HEI10 in the formation of meiotic crossovers. *PLoS Genet.* 8:e1002809. 10.1371/journal.pgen.1002809 22792078 PMC3390396

[B49] Wang X. Haber J. E. (2004). Role of *Saccharomyces* single-stranded DNA-binding protein RPA in the strand invasion step of double-strand break repair. *PLoS Biol.* 2:E21. 10.1371/journal.pbio.0020021 14737196 PMC314472

[B50] Yuan J. Chen J. (2013). FIGNL1-containing protein complex is required for efficient homologous recombination repair. *Proc. Natl. Acad. Sci. U.S.A.* 110 10640–10645. 10.1073/pnas.1220662110 23754376 PMC3696823

[B51] Zhang J. Wang C. Higgins J. D. Kim Y. Moon S. Jung K. (2019). A multiprotein complex regulates interference-sensitive crossover formation in rice. *Plant Physiol.* 181 221–235. 10.1104/pp.19.00082 31266799 PMC6716249

[B52] Zhang P. Zhang Y. Sun L. Sinumporn S. Yang Z. Sun B. (2017). The rice AAA-ATPase OsFIGNL1 is essential for male meiosis. *Front. Plant Sci.* 8:1639. 10.3389/fpls.2017.01639 29021797 PMC5624289

[B53] Zickler D. Kleckner N. (1999). Meiotic chromosomes: integrating structure and function. *Annu. Rev. Genet.* 33 603–754. 10.1146/annurev.genet.33.1.603 10690419

