# Supplementary material for: FIGNL1 Inhibits Non-homologous Chromosome Association and Crossover Formation
Source: Front Plant Sci. 2022 Jul 11;13:945893. doi: 10.3389/fpls.2022.945893 (PMC9310568; doi:10.3389/fpls.2022.945893)
Supplement: Supplementary file 1 [file Data_sheet_1.pdf]

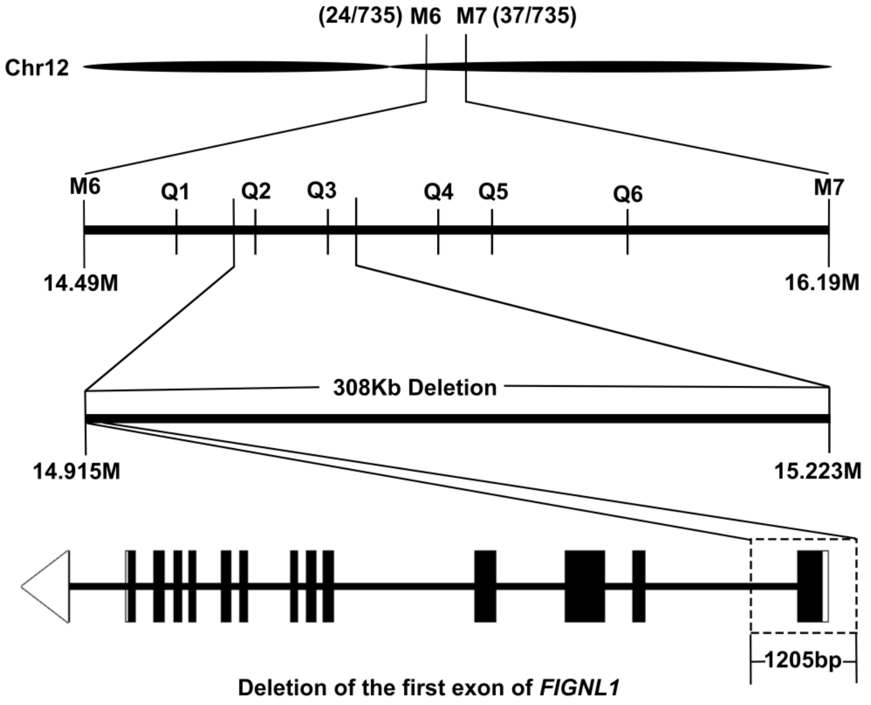

FIGURE. S1. Map-based cloning and gene structure of *FIGNL1*. Horizontal lines, region containing the mutation; vertical lines, markers. Coding regions are shown as black boxes. Untranslated regions are shown as black lines. Deleted region in the *fignl1* mutant is shown as a dotted box.

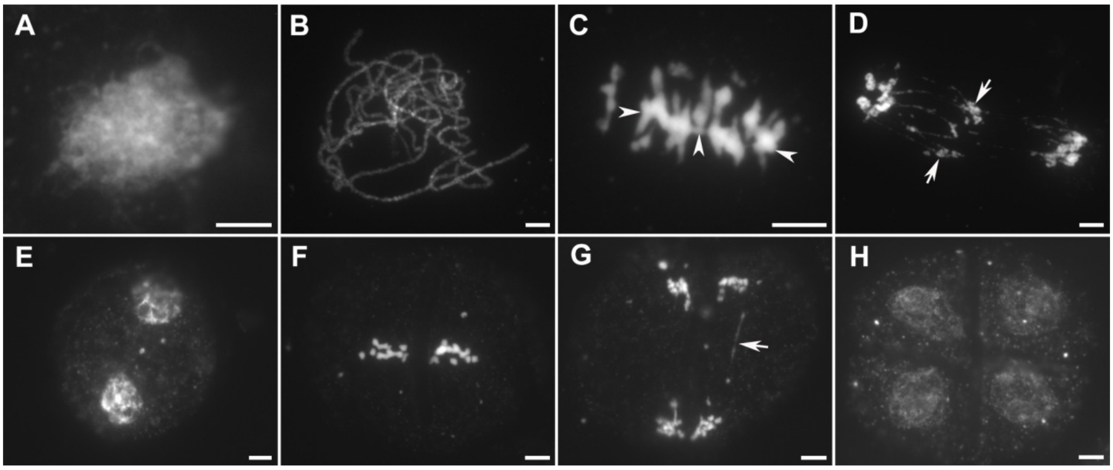

FIGURE. S2. *FIGNL1*-CAS9 plants exhibit meiotic defects similar to those in *fignl1*. (A) Zygotene. (B) Pachytene. (C) Metaphase I. (D) Anaphase I. (E) Dyad. (F) Metaphase II. (G) Anaphase II. (H) Tetrad. Arrowheads point to some chromosome associations and arrows point to bridges. Scale bars, 5 µm.

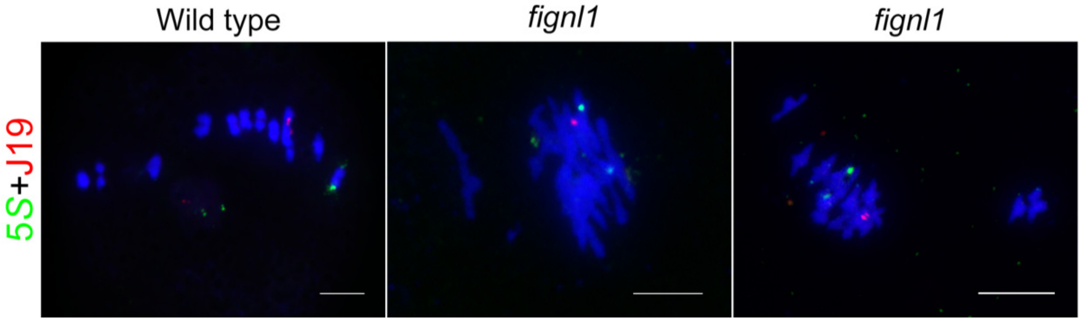

FIGURE. S3. Aberrant interaction between non-homologous chromosomes in *fignl1* occurs randomly. FISH analyses were conducted with 5*S* rDNA (green) and J19 (red) in both wild type and *fignl1*.

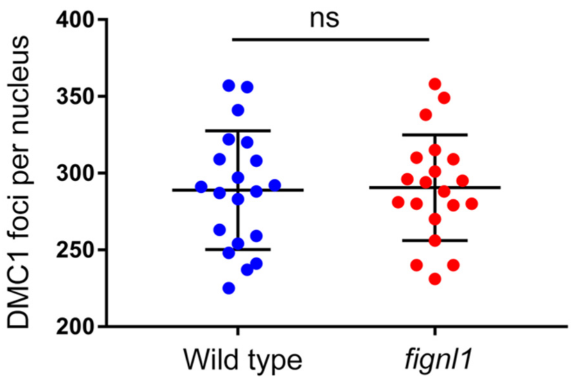

FIGURE. S4. The numbers of DMC1 foci in wild type and *fignl1*.

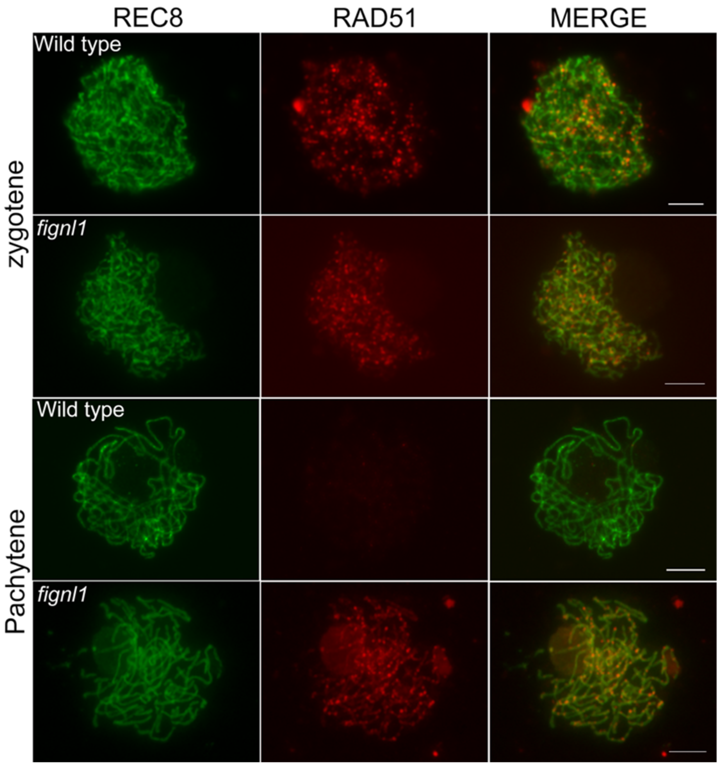

FIGURE. S5. The loading pattern of RAD51 is similar to that of DMC1 in both *fignl1* and the wild type.

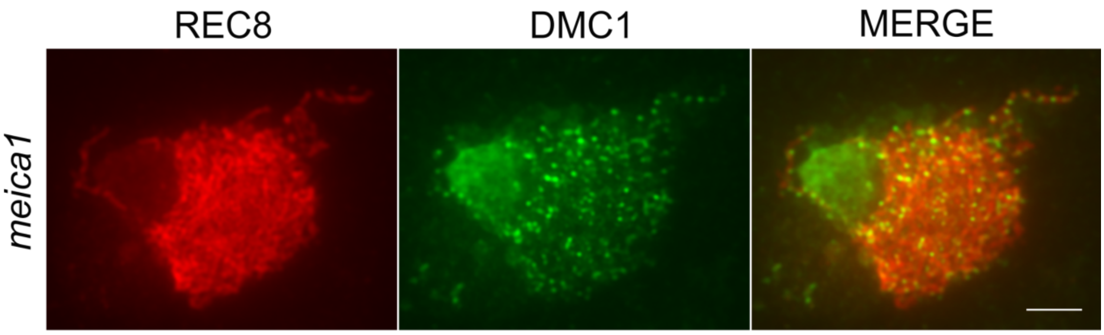

FIGURE. S6. DMC1 foci exist on pachytene chromosomes in *meica1*.

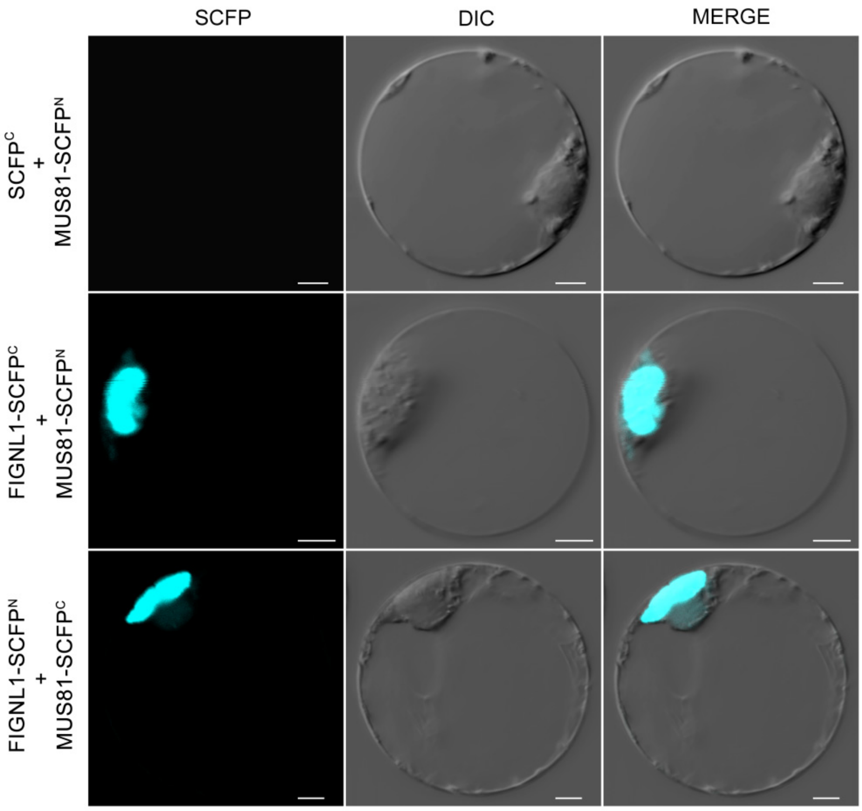

FIGURE. S7. FIGNL1 interacts with MUS81 in BiFC assays. Various vector pair combinations were cotransformed into rice protoplasts. DIC, differential interference contrast. Bars, 5 mm.

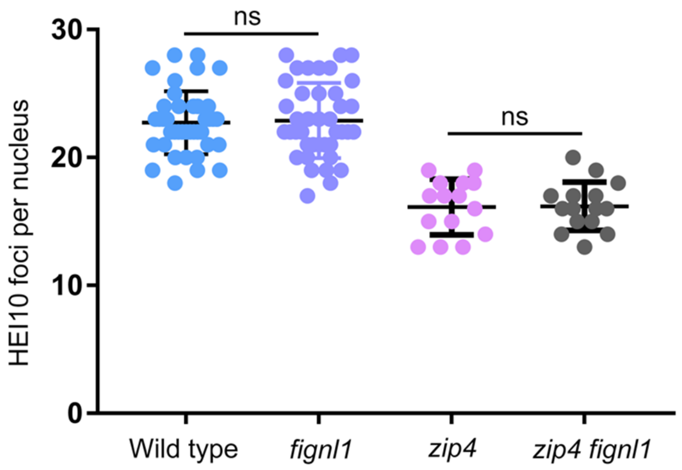

FIGURE. S8. The numbers of HEI10 foci in wild type, *fignl1*, *zip4* and *zip4 fignl1*.

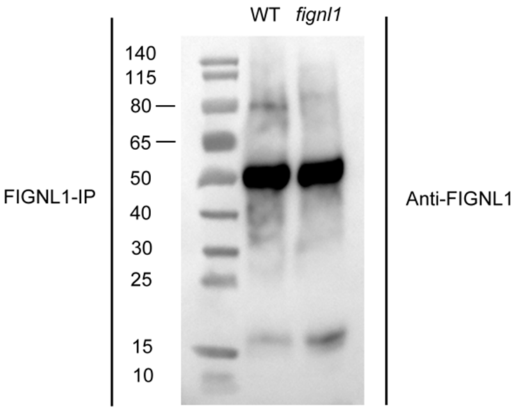

FIGURE. S9. Western blot analysis to verify the specificity of anti-FIGNL1.

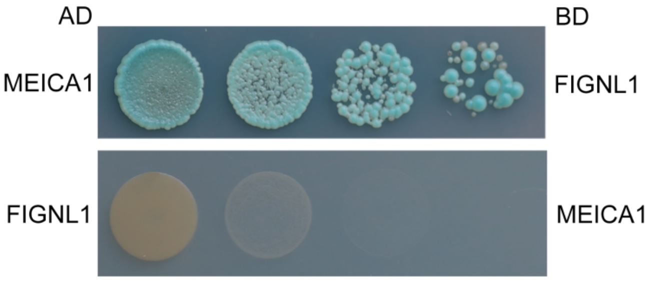

FIGURE. S10. FIGNL1 interacts with MEICA1 in yeast-2-hybrid assays.

TABLE S1. Primer sequences used in this study.

Purpose	Name	Sequence
Y2H analysis	FIGNL1-AD-F	CCATGGAGGCCAGTGAATTCATGGCGGAGCAGTCTCACGC
	FIGNL1-AD-R	GCTCGAGCTCGATGGATCCCTTAATTTGCTAAGCTCCCAA
	FIGNL1-BD-F	TGGCCATGGAGGCCGAATTCATGGCGGAGCAGTCTCACGC
	FIGNL1-BD-R	GCTGCAGGTCGACGGATCCCTTAATTTGCTAAGCTCCCAA
	MEICA1-AD-F	CATATGGCCATGGAGGCCAGTGAAATGGAGGCTGATGGTGGCGCTG
	MEICA1-AD-R	CGATGCCCACCCGGGTGGAATTTTAAATTTTGTCAGAAAAAGAG
	MEICA1-BD-F	GCATATGGCCATGGAGGCCGAAATGGAGGCTGATGGTGGCGCTG
	MEICA1-BD-R	GGTCGACGGATCCCCGGGAATTTTAAATTTTGTCAGAAAAAGAG
	MUS81-AD-F	GAGGCCAGTGAATTCATGGCGCCGGAGGCGAGGCA
	MUS81-AD-R	TCGATGGATCCCGTATTATCCTTCAGCCCAGACAA
	MUS81-BD-F	ATGGAGGCCGAATTCATGGCGCCGGAGGCGAGGCA
	MUS81-BD-R	CGCTGCAGGTCGACGTTATCCTTCAGCCCAGACAA
	RAD51A1-AD-F	CCATGGAGGCCAGTGAATTCATGTCGACGTCGGCGGCG
	RAD51A1-AD-R	GCTCGAGCTCGATGGATCCCTCAATCCTTGACATCTGCAA
	RAD51A1-BD-F	TGGCCATGGAGGCCGAATTCATGTCGACGTCGGCGGCG
	RAD51A1-BD-R	GCTGCAGGTCGACGGATCCCTCAATCCTTGACATCTGCAA
	RAD51A2-AD-F	CCATGGAGGCCAGTGAATTCATGTCGTCGTCGGGTGCG
	RAD51A2-AD-R	GCTCGAGCTCGATGGATCCCTCAGTCCTTAACATCTGTGACGC
	RAD51A2-BD-F	TGGCCATGGAGGCCGAATTCATGTCGTCGTCGGGTGCG

	RAD51A2-BD-R	GCTGCAGGTCGACGGATCCCTCAGTCCTTAACATCTGTGACGC
	DMC1A-AD-F	CCATGGAGGCCAGTGAATTCATGGCGCCGTCCAAGCAG
	DMC1A-AD-R	GCTCGAGCTCGATGGATCCCTCAGTCTTTCGCATCCATTA
	DMC1A-BD-F	TGGCCATGGAGGCCGAATTCATGGCGCCGTCCAAGCAG
	DMC1A-BD-R	GCTGCAGGTCGACGGATCCCTCAGTCTTTCGCATCCATT
	DMC1B-AD-F	CCATGGAGGCCAGTGAATTCATGGCGCCGTCCAAGCAG
	DMC1B-AD-R	GCTCGAGCTCGATGGATCCCTCAGTCTTTCGCATCCATTA
	DMC1B-BD-F	TGGCCATGGAGGCCGAATTCATGGCGCCGTCCAAGCAG
	DMC1B-BD-R	GCTGCAGGTCGACGGATCCCTCAGTCTTTCGCATCCATT
	FIGNL1-BD-F1 F2 F3	GCCGAATTCCCGGGGATCATGGCGGAGCAGTCTCACGC
	FIGNL1-BD-R1	TTATGCTAGTTATGCGGCTCATTGGCAGGGGCATCGG
	FIGNL1-BD-R2 R6	TTATGCTAGTTATGCGGCATTATTTCTGATAGGAGGAA
	FIGNL1-BD-R3 R7	TTATGCTAGTTATGCGGCTGAAGGAAGGGGAATATATA
	FIGNL1-BD-F4	GCCGAATTCCCGGGGATCGGAGGATCCACTTCCAATAT
	FIGNL1-BD-R4 R5	TTATGCTAGTTATGCGGCATTTGCTAAGCTCCCAAATT
	FIGNL1-BD-F5	GCCGAATTCCCGGGGATCTCAGAGGCACGGGCCTGGAT
	FIGNL1-BD-F6	GCCGAATTCCCGGGGATCGAGTTTGTGACTGCCAGGAC
	FIGNL1-BD-F7	GCCGAATTCCCGGGGATCTCTCCTGGAAGAGGTCTTCT
	MEICA1-AD-F1 F2	GCCATGGAGGCCAGTATGGAGGCTGATGGTGGCGC
	MEICA1-AD-F3 F5	GCCATGGAGGCCAGTGTTATCCTGCTGAACATCTC
	MEICA1-AD-F4	GCCATGGAGGCCAGTTGGAAATTTGTTCCAGTTGA
	MEICA1-AD-R1	TGCAGCTCGAGCTCGTTGAGAACCACCAGAAGATG
	MEICA1-AD-R2 R5	TGCAGCTCGAGCTCGCAGCTGAGCAAAAGACGTAC
	MEICA1-AD-R3 R4	TGCAGCTCGAGCTCGAATTTTGTCAGAAAAAGAGG
**LUC assay**	pC1300-CLuc-MEICA1-F	GCGTCCCGGGGCGGTACCATGGAGGCTGATGGTGGCGC
	pC1300-CLuc- MEICA1-R	GTAGTCCATTTGTTGGATCCAATTTTGTCAGAAAAAGAGG
	pC1300-NLuc-MEICA1-F	GGGGACGAGCTCGGTACCATGGAGGCTGATGGTGGCGC
	pC1300-NLuc-MEICA1-R	GCGTACGAGATCTGGTCGACAATTTTGTCAGAAAAAGAGG
	pC1300-CLuc-	GCGTCCCGGGGCGGTACCATGGCGGAGCAGTCTCACGC

	FIGNL1-F	
	pC1300-CLuc-FIGNL1-R	GTAGTCCATTTGTTGGATCCATTTGCTAAGCTCCCAAATT
	pC1300-NLuc-FIGNL1-F	GGGGACGAGCTCGGTACCATGGCGGAGCAGTCTCACGC
	pC1300-NLuc-FIGNL1-R	GCGTACGAGATCTGGTCGACATTTGCTAAGCTCCCAAAT T
**CRISPR-CAS9**	FIGNL1-cas9-F	GGCAGCTCCGGGGAGCCGACGAAC
	FIGNL1-cas9-R	AAACGTTCGTCGGCTCCCCGGAGC
**BiFC assay**	FIGNL1-pSCYCE(pSCYNE)-F	CCTACTAGTGGATCCATGGCGGAGCAGTCTCACGC
	FIGNL1-pSCYCE(pSCYNE)-R	AGCGGTACCCTCGAGTTAATTTGCTAAGCTCCCAA
	MEICA1-pSCYCE(pSCYNE)-F	CCTACTAGTGGATCCATGGAGGCTGATGGTGGCGC
	MEICA1-pSCYCE(pSCYNE)-R	AGCGGTACCCTCGAGAATTTTGTCAGAAAAAGAGG
	MUS81-pSCYCE(pSCYNE)-F	CCTACTAGTGGATCCATGGCGCCGGAGGCGAGGCA
	MUS81-pSCYCE(pSCYNE)-R	AGCGGTACCCTCGAGTTATCCTTCAGCCCAGACAA
**Gene mapping**	C12-M6-F	ATTCTACTCGGCAATCAGG
	C12-M6-R	ACACGTCTCCATCCCTATA
	C12-M7-F	GCTTCTGATTGTTCCCCTGAG
	C12-M7-R	AGTACCTGCACGAGTTCAACCGCCT
	C12-Q1-F	GGCACTGATTTATGGGACA
	C12-Q1-R	TGTACCGGTGAACTCCCTCT
	C12-Q2-F	TGCAGTAGAAACATGAGACA
	C12-Q2-R	TACCCTTCCATTACAGTGAG
	C12-Q3-F	GAGTTACCAGGTGCTCCCA
	C12-Q3-R	GATGTTGGCGGTTGTAGTG
	C12-Q4-F	CTTCACATTGCTGGGGACA
	C12-Q4-R	TGCCAGGACACTCGTATTTTA
	C12-Q5-F	TGCTCCTAAATTATTTTATCCTC

	C12-Q5-R	CTAAAGGGTAAAACTGATACAAA
	C12-Q6-F	GGCTACGGTGGTTACAAA
	C12-Q6-R	CCGTCATTTGCCAAGAT
	m96-tail-inner	CTAAACTTGAGTCAAGGTCT
	m96-tail-outer	CAGTACGCAATGTGCAACAT
**Cloning by 5′ RACE**	5′RACE-RT	GCAGCATGTCCTTTGGATTTCT
	5′RACE-S1	AGGTCAGACAGTGCTGTGTT
	5′RACE-R1	GCCGAACTGGAGGGAGTGGA
	5′RACE-S2	GCAAAGACAGGAGAAGTT
	5′RACE-R2	CTATCGTCCGCCTCCTTCCT
**Cloning by 3′ RACE**	3′RACE-1F	TGGATCTTGAAGATGATGACAG
	3′RACE-2F	AGTAATGATGAAGCCGATGCC
